# Love Is Blind! Exploring the Impact of Brand Love on eWOM in Chinese Hospitality Industry

**DOI:** 10.3389/fpsyg.2022.916206

**Published:** 2022-06-29

**Authors:** Muhammad Bilal, Umair Akram, Yunfeng Zhang, Shukai Cai, Zhuliang Wang

**Affiliations:** ^1^School of Economics and Management, Anhui Polytechnic University, Wuhu, China; ^2^School of Business and Management, RMIT University, Ho Chi Minh, Vietnam; ^3^School of Hotel Management, Guilin Tourism University, Guilin, China

**Keywords:** eWOM, brand love, information quality, system quality, reward, virtual interactivity

## Abstract

The rising penetration and value of online information reviews in the hospitality industry have been extensively examined. People are increasingly interacting on interactive online media, prompting firms to create online communities to share electronic word-of-mouth (eWOM) with them and with one another in order to increase brand love. This study seeks to discover what drives consumers to participate in these online brand communities. To examine the research model, an online survey was conducted on 508 consumers who had shared eWOM on social networking sites (SNS). Structural equation modeling (SEM) was employed to test all hypotheses. The findings show that each of the variables has a high impact on brand love, with information quality and virtual interaction having the most significant impact. As a result of brand love, eWOM is significantly increased. Additionally, findings show that the gender gap in the online setting is increasing, as the influence of all characteristics then reward of online brand communities on brand love was adaptable among both male and female associates. Considering the effects of online features (information quality, system quality, virtual interaction, and reward) on online brand communities' impact on brand love and eWOM. Online hotels manager is advised to carefully develop their marketing strategies to retain and attract new consumers. Furthermore, online hotels manager can use the findings from this study to understand the consequences when eWOM is strongly influenced by online brand communities' features. This study is one of the first to illustrate and empirically confirm insight into how online features affect brand lovers in online brand communities. The study adds to the body of knowledge concerning the effectiveness of social media marketing in the Chinese hotel sector.

## Introduction

In recent years, brands and firms are adopting social networking sites (SNS) as the newest way to discuss with potential and actual consumers, as well as to upgrade hotel services (Hajli and Sims, 2015; Hu and Kim, 2018). SNS is the interactive channel that enables consumers to connect with their brands regularly (Tafesse, 2016). According to recent statistics, the number of SNS users was 71%, and it is predicted that by 2020, the number of active SNS users will be more than 3 billion (Statista, 2018). Notably, SNS users in China have increased to 61.7%, and daily users of SNS have reached 57.9% (Zong et al., 2019). SNS has become a powerful communication platform by which hotel firms interact with consumers. Thus, a significant objective for brands is to raise consumers' attachment through their SNS by analyzing relevant consumer segments that will be most flexible to their communication through their SNS (Grange and Benbasat, 2018).

The fast expansion of SNS has changed how information is accessed and delivered to consumers with an online platform wherein they can easily share information and suggestions about a brand or experience, namely electronic word-of-mouth (eWOM) (Kim et al., 2018; Bilal et al., 2022). eWOM has become an essential platform for several firms because it influenced consumers' behavior and attitude (King et al., 2014). Specifically, these impacts are influential in the service firm. In the scenario of the intangible and experiential nature of the offering, consumers are motivated to seek additional information from experienced consumers who have absorbed the service (Papathanassis and Knolle, 2011). Tourists strongly depend on eWOM when making trips (Zhang et al., 2014). Approximately 90% of visitors read eWOM before scheduling trips (Erskine, 2017). Online consumers' reviews provide assessments in terms of eWOM about services, products, and firms on third-party websites. Online consumers' reviews strongly influence fostering eWOM (Gottschalk and Mafael, 2017; Bilal et al., 2020a,b). According to Phillips et al. (2017), eWOM is a significant source of information for the hotel sector to understand their consumers' desires and enhance their hotel booking and accommodation quality.

It is particularly true in the hotel firms in China, where online information has been considered a reliable and significant innovation in the recent decade (Gössling et al., 2016; Hu and Kim, 2018). Online communication forums such as SNS and hotel websites have become important quality information sources for consumers and the hotel industry (Liu and Park, 2015). Consumers post and seek information from this platform because they think it is reliable, credible, and independent and can decrease the chances of their uncertainty (Schuckert et al., 2015). For industries, online information is a means to figure out the consumers' perceptions and increase their performance (Phillips et al., 2017). Therefore, the quality of online hotel information influences the consumers' choices and leads to brand love (Audrezet and Parguel, 2018).

The present research attaches to the attribution theory (Heider, 1958). The theory has examined the outcomes of the online community posting information on consumers' hotel choices and booking intentions. Based on attribution theory, people are pretty susceptible to virtual environment inputs. Accordingly, when consumers discover various online hotel information, they are able to attach specific hotels (in terms of information quality, system quality, virtual interactivity, and rewards), which eventually influence the hotel booking intention.

The current study focuses on SNS in terms of the online community, which examined the fact that the online community platform provides an immense opportunity to share opinions or evaluate hotel service providers. The significance is also encouraged by the undeniable fame of the forum. In the marketing literature, the online community's desire for brand love research is broadly approved (Hollebeek et al., 2014). Significant attention has been paid to online community research on brand love in recent years, but the field of empirical exploration is still underdeveloped (Keh and Sun, 2018). Research on brand love motivation in online groups has lagged behind the industry's ever-changing context despite this rational demand. Earlier research has indicated the need to explain online community characteristics and their influence on brand love (Keh and Sun, 2018) because these characteristics consider a consumer's complete impression of the online community. Multiple studies have demonstrated the impact of online community qualities on commitment, fulfillment, and brand awareness (Jang et al., 2008). Regardless of the fact, studies analyzing the online community characteristics and the direction in which these variables elements brand love is uncommon.

Several important contributions are made by this study to the existing literature. First, we propose and empirically examine a conceptual framework for the development of brand love and eWOM *via* online community characteristics (information quality, system quality, virtual interactivity, and reward). Second, whether the online comments generated on SNS influence hotel booking intention; third, this study investigates the role of gender as a moderator in the relationship between information quality, system quality, virtual interactivity, and reward. Finally, our study support hotel industries in developing an effective plan to reduce uncertainty and encourage consumers to read online hotel reviews for hotel booking. The research findings will have practical implications for the Chinese modern hospitality industry.

## Theoretical Development

### Electronic Word-of-Mouth in the Hotel Industry

The eWOM is a significant source of information because it provides experience-based, completed, non-commercial, and up-to-date information online (Hennig-Thurau et al., 2004). eWOM is extended in different practices, such as social media (e.g., WeChat), online communities, and online information can be stored and revised on the web. eWOM primarily transforms the spatial, expressive, and private social network limits of traditional WOM and investigates the impact of a diverse scope of more powerful consumers than family, peers, or relatives in the offline scenario (Dellarocas, 2006). eWOM offers some rare kind of feature. First, eWOM extends the space of influence because eWOM is easily non-perishable and referable nature. Second, SNS helps consumers to share and search for information as well as to manage online transactions. Based on this eWOM has become an essential channel in consumers' hotel booking and decision-making behavior (Ye et al., 2009). Because many hotel websites give online opportunities where consumers to share information and communicate with hotel experience consumers. An investigation by Tsao et al. (2015) shows that 87% of consumers believe that online information helps them book hotels confidently, and 98% evaluate that online information is accurate.

In the context of hotel industries, eWOM can get multiple forums such as posting and hotel reviews on the consumer suggestion sites (e.g., official hotel websites), revealing to be incredibly imperative (Phillips et al., 2017). These reviews contain descriptions and hotel services, and usually, these reviews are created by hotel staff. With the rising trend of online electronic communication, young consumers are always eager to post reviews of their visits on hotel websites (Law et al., 2018). Therefore, hospitality consumers are encouraged to evaluate the analysis, comments, and shared experiences of other customers online before making a reservation. According to the study's literature, the tourism sector has a massive impact on eWOM, and hotels appear to be the most concerning. Consequently, this area calls for considerable attention (Ladhari and Michaud, 2015).

### Attribution Theory

In line with (Heider, 1920), attribution theory serves as a foundation for the following theories on peoples' views advised by multiple writers. The goal of that study was to resolve the conflict between real objects and fleshly information. Far ahead, Haider focused on the domain of social interaction and measured how people create a feeling for other people. Found by Heider (1958), “persons are recognized as action places and as such possible do something to us. We may positively or negatively influence them, and they can be beneficial or harmful to us deliberately. Persons have capabilities, aspirations, and opinions; they can manifest decisively and might notice or observe us.”

Attribution Theory is defined as: “a theory that explains the cognitive process by which individuals assess the causes of behaviors and events in their globe” (Mullen and Johnson, 2013, p. 174). Haider's attribution theory proposes a condition of the psychology of interpersonal associations (Patwardhan and Patwardhan, 2016); additionally, Haider expanded the theory by concentrating on the people's different responses (Heider, 1958).

The present study, attribution theory, is recognized as a core theory that connects hotel information quality *via* online and brand love, coined the term “attribution theory,” which appeared from the psychology of personal associations. This theory targets the person's opinion on an event and its result on peoples' attitudes (Heider, 1958). “Attribution theory behaves with how people clarify behavior and events based on their casual judgment. Their explanations play a vital role in deciding reactions to these experiences or behavior (Chang et al., 2015, p. 50). In these circumstances, when consumers are disclosing the information, they try to compare information in the brand offering style. As a result, satisfaction requires information that may lead to a relationship with a particular brand or brand lover (Chakraborty and Bhat, 2018); brand lover likes to share and recommend to other online users as a result of increasing the number of brand lovers as well as their impact on consumer hotel booking behavior (Jackson, 2019).

### Brand Love

Proposed by Rubin (1973), brand love is a mindset that consumers hold toward a specific brand, which adds their skill to feel, behave, and think concerning the particular brand. Brand love was investigated to influence competitor brand-related opinions such as brand relationships and eWOM. An individual ability to recognize and desire a few brands is one of the decision variables so that consumers feel willing to buy a particular brand continuously. The relationship it has also been recognized to develop brand love is enduring and profound; thus, brand love provides unique qualities (Albert and Merunka, 2013). Carroll and Ahuvia (2006) defined it as “the level of feelings and dedicated connection that a satisfied consumer has to a specific brand.”

Furthermore, Batra et al. (2012) argue that brand love is emotionally attached to continuous interaction with a specific brand. From SNS's perspective, brand love may develop with self-expressive brands on SNS, considering a rewarding and favorable interaction relationship that they have “liked” (Wallace et al., 2014). For example, consumers who love a particular brand are eager to pay for their brands and are excited to share, collect, and suggest the products or brands.

Recently, brand love has received devotion in the context of the hospitality research concept because the setup has been expressed to influence key marketing variables such as brand information quality, willingness to award a premium price, spreading eWOM, switching resistance, and repurchase intention (Batra et al., 2012; Wang et al., 2019). Moreover, brand love and changing resistance loyalty highlight the literature that brand love is crucial to the hotel business, which has severe issues in holding loyal consumers. Similarly, the electronic marketing of multiple services, such as hotels, travel packages, cruises, and flights, has been increased because of various benefits to E-tailers and consumers. SNS has been established as the essential channel for hotel firms during the past decade. In addition, SNS extends eWOM influence on consumers deciding on hotel brands. SNS provides the opportunity to acquire other consumers' hotel experiences (Su et al., 2015; Gleason et al., 2019).

## Research Model and Hypotheses

### Information Quality and Brand Love

The previous multiple studies have applied various measures of information system success. These measures involve users' specific brand information (Gao et al., 2017), improved performance and decision quality (Mpinganjira, 2016), and information systems benefits (Peters et al., 2016). All these different studies are given a valuable point of information quality in an online community hotel appreciably on the information quality connected to the brand. In hotel industries, information quality is considered as a valuable asset that has been studied by many research scholars (Holbrook, 2006). Information quality in an online context is defined as “the quality of the information displayed on a website as perceived by users” and reviews the differentiation between users' perceptions and expectations of information circulation (Liu et al., 2018). From an online viewpoint, consumers perceive information quality as the expansion to which they provide information to verify their potential and fulfill their required information that may lead to a relationship with the specific brand. High-quality information allows consumers to obtain information and receive a useful opinion on one particular brand (Zheng et al., 2013). When an online consumer gets a more outstanding quality of information, they consider it likely valuable (Kim et al., 2016). In line with Albert and Merunka (2013), they demonstrated that higher quality of information might have a vital role in developing brand love. Therefore, we suggest that:

H1. Information quality positively influences on brand love of hotel firms promoted on SNS.

### System Quality and Brand Love

System quality indicates “convenient and fast seeking information in an online platform” (Nikhashemi et al., 2017). It is “an evaluation of the certain extent to which the system is designed to ease of use technically, user-friendly, error-free, well documented, response time, resilience, and security.” A robustly considered system is vital for obtaining hotel benefits such as increased revenue, enhanced process performance, and cost reduction. Conversely, a poorly designed system may turn out destructive to hotels and lead to intensive service costs and low hotel efficiency (Moody et al., 2017). In addition, the quality of system factors is influencing because they reveal to shaping the service procedure to be more pleasant and energetic (Langaro et al., 2018). System quality gives an effective impression to its consumers to reply positively to the brand's visible view, develop a relationship with the brand, convince repeat buying, and develop trust (Shin et al., 2013). Consumers like to participate in a high-quality system, recall the brand, and share positive eWOM (Gorla et al., 2010). According to Albert and Merunka (2013), consumers connecting their relationships could have a great act in developing brand love, and system quality may also play a vital role in brand love. Therefore, we postulate the link:

H2. System quality positively influences on brand love of hotel firms promoted on SNS.

### Virtual Interactive and Brand Love

Theory suggests promising to react and multimedia web characteristics in developing multiple dimensions of relationship building with consumers in the context of virtual environment research on interactivity (Di Pietro et al., 2012). This research shapes definition of Steuer's (1992, p.4), which virtual interactive (VI) defines as “the extent to which online users can engage in modifying the website's content in real-time.” This study defines VI as SNS users and hotel industries discussing directly with each other, without regard for time or distance, sharing, investigating, and providing hotel-related information via SNS in a timely manner. According to this definition, interactivity refers to the value of tools that promote interactivity with and between consumers and encourage consumers to share information on the hotel's website (Jakeli and Tchumburidze, 2012). Numerous researches on VI investigate the abrupt influence on the creation of healthy ranks of branding elements. However, the improvement of consumers' relationship with the brand has not yet been examined (Jakeli and Tchumburidze, 2012), specifically in the hotel (Xiang et al., 2014). People, who are more attached to the brand, search for information about the brand, are involved to a higher degree in the choice between alternatives, are well familiar with the difference between similar categories of the brand, and show more preference for the exact brand. VI plays a vital role in developing brand trust and satisfaction. Interactivity encourages consumers to join and stay in SNS. According to Barreda et al. (2016), they argued that VI positively influences brand love in a hotel context. Therefore, we postulate the link:

H3. Virtual interactivity positively affects the brand love of hotel firms promoted on SNS.

### Reward and Brand Love

The monetary or psychological rewards pattern for devoted SNS users is interpreted as the reward for activities (Kim et al., 2013). In the hotel industry, rewarding is one of the main elements that develop a relationship with a specific brand (Barreda et al., 2016). In this article, reward indicates the level of monetary or psychological motivation for its consumer and reflects all the favor that consumers receive through their relationship with a specific brand (Newman and Sheikh, 2012). The reward can include financial (discounts, special deals, and loyalty) value, functional (moral support and right information) profits, social (image building, appreciation, and altruism) benefits, and psychological (entertainment and belonging) benefits (Wirtz et al., 2013).

Offering various incentives is indicated as an essential driver for developing a consumer's relationship with a specific brand and service (Rohm et al., 2013). These incentives influence consumers' decisions to choose a particular brand from a competitive platform and to join such communities for development purposes. Past research found that rewards are positively related to the level of consumer engagement (Wirtz et al., 2013). Similarly, Lee et al. (2021) demonstrated that hotel industries need to allot psychological and financial rewards to develop consumers' relationships, interest in, and preference for a brand/service. Therefore, we postulate the link:

H4. Reward positively affects brand love of hotel firms promoted on SNS.

### Influence of Brand Love on eWOM

The direct relationship between brand love and eWOM is well documented in the literature (Aro et al., 2018). According to Leong et al. (2020), eWOM plays a great role in influencing consumers' behavior. If the consumers are intensely engaged with the brands, they like to show their feelings toward it by informing and suggesting to others about it (Leong et al., 2020). Independently spend brand-related effort/time publicly spreading their feelings on different SNS (Hollebeek, 2011). Thus, consumers are considered loyal brand activists who like brands on social sites. Brand loving motivates the consumers to suggest it to their family and friends.

Searching brands on SNS give the brand a chance to interact with consumers who have an emotional link with the brand and join with the brand by high association and spread eWOM (Bilal et al., 2021). Brand lover consumers like to discuss it through the construction and identity process (Batra et al., 2012). The effect of brand love on eWOM has been positively disclosed (Carroll and Ahuvia, 2006). The current study suggests that consumers who like a brand on SNS appear as the evangelists for the product/brand, taking into sharing eWOM. These brands dearest not only interface with others internally, they similarly have passionate holding with the brand ground on which they plan to share the brand with others (Leong et al., 2020). Therefore, we postulate the link:

H5. Brand love positively influences the eWOM of hotel firms promoted on SNS.

### Gender as a Moderator

Apart from gender-specific skills, the theory of gender-based socialization shows that men and women who are indifferent to specific cultural atmospheres are able to achieve personal attributes and individual views of different sexes, giving them the ability to be implored by masculine or feminine disposition (Barry et al., 1957). Consequently, propelled by different values and options in the ethical area, both genders cultivate value-sets, respectively (Mason and Mudrack, 1996). Discrepancies between the two genders have been handled by two mainstreams in the research area, which are the biological sex research stream (Chang, 2006; Bilal et al., 2020a,b) as well as the gender identity research stream (Gould and Weil, 1991).

For one thing, men and women are reckoned by the former one throughout the field of gender regarding biological sex (Kolyesnikova and Dodd, 2009). Nevertheless, the latter is prone to concentrate on the psychological sex identity when it comes to gender (Gould and Weil, 1991), which is built on two contrasting personality traits of gender. Researchers discover that it is a gender identity that exerts influence on customers' views and simultaneously plays a role in predicting behavioral performance in consumers' activities.

Nonetheless, the part gender identity plays in the bounds of consumer behaviors research is suspected (Kolyesnikova and Dodd, 2009), and in the meantime, compared with gender identity, researchers have adopted biological sex as a vital factor in predicting behaviors of consumer groups. Additionally, from a realistic perspective, recent studies have increasingly unveiled men and women as a separation variable (Das, 2014). Therefore, this study considers gender to be a biological reality (men and women).

A good quantity of research has evaluated the role of gender in the marketing and management domain, but such studies are rare in association with the online situation (Ladhari and Leclerc, 2013). According to online research, gender differences in information and decision-making processes between men and women play an essential role in using and adopting the internet by men and women (Verhagen and Van Dolen, 2011). As opposed to women, men engage in fewer trials and exploratory behavior, prefer web booking and have optimistic beliefs about web advertisement as opposed to traditional media (Wolin and Korgaonkar, 2003). The moderation result regarding gender in the online environment is questionable. According to one research direction, the gender gap performs a key position in the online context because men and women interact contrarily on the web (Verhagen and Van Dolen, 2011). Another research direction proposes that online media's gender gap reduces as both men and women find critical features like system quality and information quality, etc., as equally essential (Liu et al., 2018). Furthermore, perception and similarity among men and women are found in how online experience and web atmosphere influence their SNS consumers' behavior, attitude toward SNS, and hotel-quality service. As a result, this research aims to recognize this discrepancy by examining how different sexes establish links between the critical feature of online reviews and brand love.

Men and women express different perceptions, behavior, and attitude toward online-based interaction (Chen and Macredie, 2010). Past studies reveal that men tend to pay attention to the detailed information that they consider essential. On the other hand, women adopt relational processors who review all existing information and explore the relationship and differences or similarities among different kinds of information (Meyers-Levy, 1989). These findings suggest that:

H6. Information quality and brand love vary according to gender.
H7. System quality and brand love vary according to gender.
H8. Virtual interactivity and brand love vary according to gender.
H9. Reward and brand love vary according to gender.

## Methodology

### Research Setting

**Figure 1:**
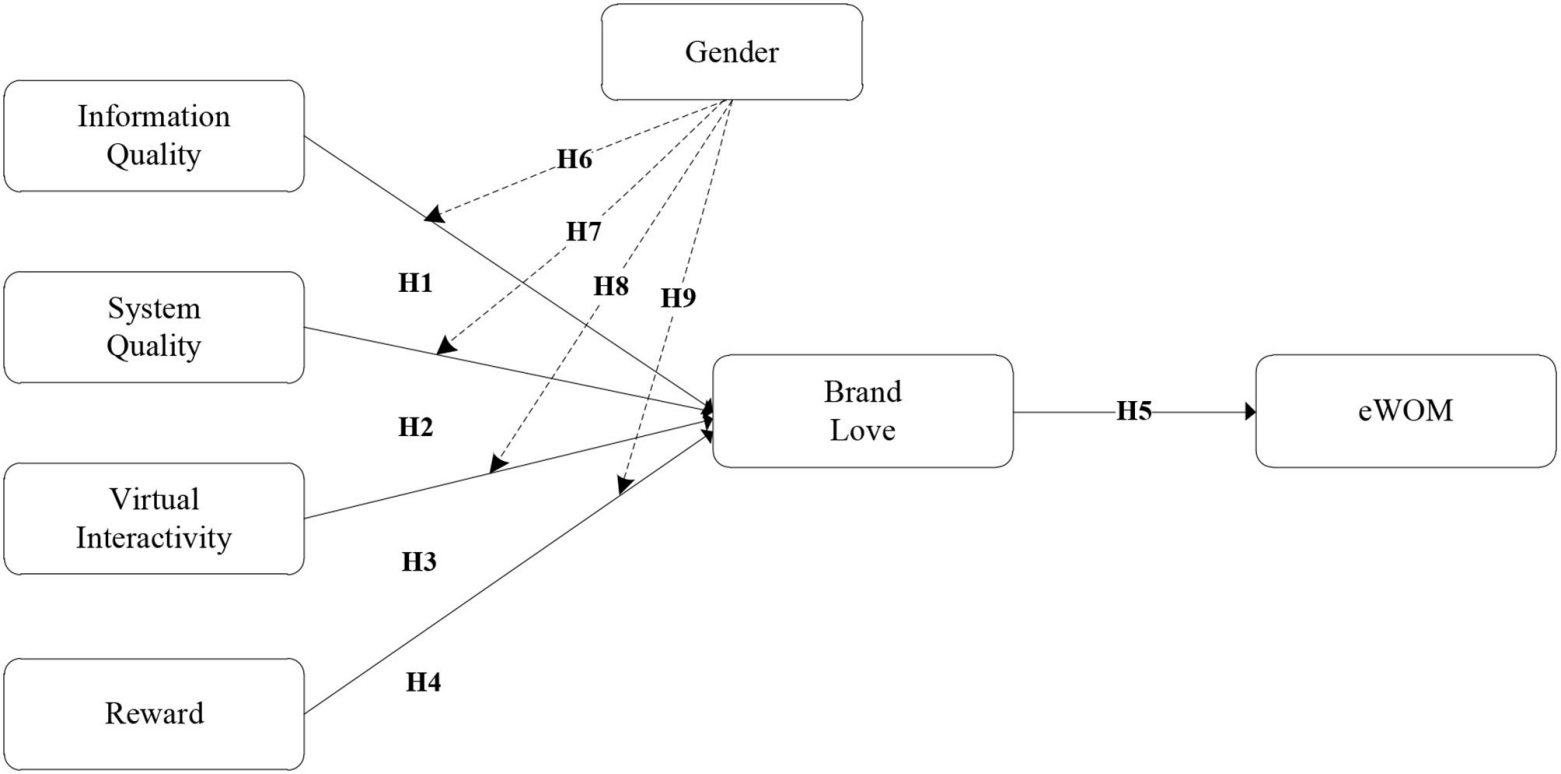
Research model.

As an appropriate research framework (see Figure 1), this study was conducted in Beijing because China hotel market is the fastest-growing market in the world. In 2016, a number 59.27 million of people stayed in hotels overnight (Business Wire, 2016). The hotel industry in China has 10,956 hotels, with 807 five-star hotels. Hotels industries in China had made ≈211.278 billion CNY, of which 42.86% was related to restaurants, and room-related services accounted for 35.55%. The average rate of a one-night hotel room in China was CNY357.61 in 2016, and the average residence was 55.59%. In 2014, China hotel industry provided more than 66 million job opportunities (Travel, 2015). In addition, the administration of China is working on a sustainable strategy to motivate the local hotel industries in order to promote hotel chains that will provide fresh jobs and stable the economic gap between rural and urban areas and between upper and middle classes. Considering the world's largest population group, the attitude of Chinese consumers regarding consumption is shifting every day. Therefore, the government of China developed a choreographed plan that motivates the hotels' industries to expand and improve, which will support the development of technology, human resources, and capital. Consumer psychologists have hypothesized the customers' perceptions of online hotel information in hospitality research, and China hotel market has become an essential field for a researcher who has assessed the behavior of online hotel consumers (Wang et al., 2015; Ahmad and Sun, 2018). We, therefore, follow this instruction and include an empirical study of consumers' psychology, hospitality, and service management.

### Sampling and Data Collection

We targeted people who had stayed in a 3-star hotel or a 4-star hotel in Beijing or planned to do so; our targeted population is Chinese. The 3-star and 4-star hotels were our first choice because they account for 70.13% of all hotels in China (Travel, 2015). According to Ahmad and Sun (2018), there is no actual method of conveying star rankings to hotels, so a European or Asian hotel with a similar star category differs from a U.S. hotel with varying star ratings in terms of the level of amenities and services. In China, the Tourism Bureau and the National Tourism Administration control the hotel rating system instead of a third-party platform such as Expedia and TripAdvisor (Su and Sun, 2007), which gives a rating based on the consumers' views, like that a more excellent rating is not the best indicator of hotel service quality. According to the UNWTO (2014), report 84% seeker *via* online information, and 75% of consumers considered hotel classification.

Similarly, 36% consumers use online information for searching the hotel brand, 52% consumers used online information to find the hotel classification (Statista, 2017). We chose 3- and 4-star hotels with higher consumer prospects than other hotels. We chose Beijing because it attracts visitors from all around China, increasing the generalizability of our findings.

Data were obtained through convenience sampling. This procedure is investigated by Seckler et al. (2015) when the questionnaire's items are suitable and when the study's nature is exploratory and relevant to the respondents. This method has been adopted in the extant literature and has been simultaneously shown as suitable for data collecting (Román, 2007). According to Seckler et al. (2015), in exploratory studies with items that are correlated to respondents, this method is reasonable and adequate. The constructs of our study have not yet been explored in the literature. Hence, the research must be exploratory, and the items on the questionnaire are applicable to the respondents, so the study meets both of these criteria. Data were collected between Augusts and November, 2021. Respondents in the hotel were contacted and asked if they were scheduled to stay (arrivals) or stayed (departures) at any 3-star or 4-star hotels in Beijing, China. We hired 6 PhD applicants to conduct the survey. Consumers accomplished the survey in ≈15 min, after which we showed sincere gratitude with cards for their support. Total, 589 eligible members were requested to contribute to the survey, and 508 responses were received for a rate of 86%. A total of 79 eligible members were not included for further analysis because of exciting values (e.g., all fives or all ones) incompleteness.

## Data Analysis

### Demographics of Respondents

Table 1 displays the demographic information for the survey participants. The mainstream of respondents was between 26 and 30 years old. Men made up 48.6% of all participants, and women were 51.3%. Approximately 67% of those polled were single. More than half of those polled had a bachelor's passed, with 40.7% having a master's degree. We classified respondents based on their online hotel booking and review experience. The mainstream of participants, 54.9%, had used social media websites. With online hotel reviews, 60.2% were moderately experienced, while 39.7 were fully experienced. Furthermore, 53.3% of participants frequently share eWOM about their hotel experience on social media.

**Table 1:** Respondents' profile (*n* = 508).

**Demographics information**	**Frequency**	**Percentage**
**Gender**		
Male	247	48.62
Female	261	51.38
**Marital status**		
Single	343	67.52
Married	165	32.49
**Age**		
20–25	71	13.97
26–30	269	52.95
31–35	59	11.61
36–40	77	15.15
Over 40	32	6.30
**Qualification**		
High school	53	10.43
Higher secondary school	49	9.64
Bachelor's degree	162	31.88
Master's degree	207	40.74
Doctorate	37	7.28
**Online hotel booking information source**		
Travel websites	61	26.9
Search engines	73	12.00
Hotel websites	95	18.70
Social media	279	54.92
**Online hotel reviews and hotel booking experience**		
Fully experienced	202	39.76
Moderated experienced	306	60.24
**eWOM experience: How often did you post hotel experiences online?**		
Never	27	5.31
Few (1–2 times)	51	10.03
Frequently (2–3 times)	159	31.29
Often (more than three times)	271	53.34

### Instruments

All of the items used in this study were adapted from the previous studies. We measure items that had been used and validated in an earlier study, with proper modifications in wordings to suit our research setting. A ten-item measure for information quality was modified from Teng et al. (2014). Six-item measures for system quality were amended by Ahn et al. (2007). Four items measured for virtual interactivity were adapted from Jang et al. (2008). A two-item measure for reward was adapted from Jang et al. (2008). Four-item measures for brand love were adapted from Carroll and Ahuvia (2006). A four-item measure for eWOM was adapted from Alhidari et al. (2015). All measurement items were taken on the five-point Likert scales, from 1 “totally disagree” to 5 “totally agree.”

### Analysis and Results

The first step was to analyze the data; we checked all the variables outliners, missing values, the accuracy of data, multicollinearity, and normality. The next step was the Confirmatory Factor Analysis (CFA) to check for convergent and discriminatory validity by AMOS 20; the psychometric character of the scales used. Furthermore, the validity of the content and professional recommendations was removed, as were items with low (0.5) factor loading. Structural Equation Modeling (SEM) was used to test the model and presented the hypothesis of this research.

Table 2 shows the construct's factor loading, Composite Reliability (C.R.), Average Variance Extracted (AVE), and Cronbach a. Each item's loading was larger than 0.5, so convergent validity standards were fulfilled (Bagozzi and Heatherton, 1994). All constructs' value of the Cronbach's were suitable (>0.7). The C.R. must be above 0.70 (Fornell and Larcker, 1981); it ranged between 0.81 and 0.93, thus satisfactory. The constructs' AVE ranged from 0.61 to 0.79, which was higher than the accepted value of 0.50 (Fornell and Larcker, 1981). Podsakoff et al. (2003) examined the common method bias; Harman's single-factor test was utilized. The findings show that the initial factor was 28%, below 50% of the whole variance; all the loads showed considerable *t*-values (*p* < 0.01). Consequential convergent validity (>0.5), which represented the common method bias, did not pose a threat to our research. Discriminant validity is defined as “the level to which various ideas are unique from one another” (Bagozzi and Heatherton, 1994, p. 20); in this study, the AVEs square root of each item is compared to all of its relevant associations (Fornell and Larcker, 1981) (see Table 3).

**Table 2:** Reliability and convergent validity.

**Constructs**	**Items**	**Means**	**SD**	**Item loading**	**CR**	**AVE**	**Cronbach's**	**KMO**
Information quality	10	3.32	1.17	0.79–0.89	0.81	0.72	0.82	0.83
System quality	6	3.52	1.14	0.81–0.87	0.89	0.79	0.92	0.76
Virtual interactivity	4	4.11	1.31	0.88–0.93	0.91	0.67	0.86	0.82
Reward	2	3.20	1.15	0.80–0.91	0.86	0.78	0.89	0.75
Brand love	4	3.15	1.10	0.76–0.94	0.89	0.61	0.87	0.79
eWOM	4	4.35	1.73	0.77–0.95	0.93	0.65	0.91	0.84

**Table 3:** Discriminant validity.

**Constructs**	**1**	**2**	**3**	**4**	**5**	**6**	**Square root of AVE**
(1) Information quality	–						0.84
(2) System quality	0.44	–					0.88
(3) Virtual interactivity	0.38	0.43	–				0.81
(4) Reward	0.32	0.31	0.47	–			0.88
(5) Brand love	0.34	0.37	0.49	0.37	–		0.78
(6) eWOM	0.37	0.39	0.39	0.38	0.43	–	0.80

### Structural Model

To estimate the model fitness, assessments containing the χ^2^ statistics, root mean square error of approximation (RMESA), the goodness of fit index (GFI), normed fit index (NFI), and comparative fit index (CFI) were evaluated. As per Joreskog and Sorborm (1989), appropriate values for the NFI and GFI are >0.9; the CFI, according to Hu and Bentler (1999), should be below 0.95. The RMSEA values <0.06 show an acceptable range (Browne and Cudeck, 1993). Table 4 shows the structural model result that information quality (β = 0.51; *t* = 5.63, *p* < 0.05), system quality (β = 0.54, *t* = 4.64, *p* < 0.05), virtual interactivity (β = 0.47, *t* = 5.32, *p* < 0.05), and reward (β = 0.52, *t* = 4.43, *p* < 0.05) display significant positive effects on brand love, with virtual interactivity and information quality showing the solidest effects. The results also indicate a significant positive effect of brand love on eWOM (β = 0.54, *t* = 0.52, *p* < 0.05). Thus, H1, H2, H3, H4, and H5 are supported.

**Table 4:** Hypotheses testing results.

	**Hypotheses**	**β**	***t*-values**	**Results**
*H1*	Information quality → Brand love	0.51	5.63	Accepted
*H2*	System quality → Brand love	0.54	4.64	Accepted
*H3*	Virtual interactivity → Brand love	0.47	5.32	Accepted
*H4*	Reward → Brand love	0.52	4.43	Accepted
*H5*	Brand love → eWOM	0.54	5.52	Accepted

This research conducted a multi-group analysis in AMOS 20.0 to check the moderating influence of gender as expected in H6, H7, H8, and H9. The whole sample was divided into two sets, male (*n* = 247) and female (*n* = 261). For each group, the independently constructed model fit well: for men, χ^2^ = 275.141, *df* = 127, χ^2^/*df* = 2.16, *NFI* = 0.911; *CFI* = 0.925, *IFI* = 0.919, *GFI* = 0.922, *RMSEA* = 0.059; for women, χ^2^ = 239.51, *df* = 137, χ^2^/*df* = 1.74, *NFI* = 0.907; *CFI* = 0.915, *IFI* = 0.909, *GFI* = 0.911, *RMSEA* = 0.072. A solid model fit was validated again by the structural multi-group: χ^2^ = 299.243, *df* = 139, χ^2^/*df* = 2.152, *NFI* = 0.903; *CFI* = 0.923, *IFI* = 0.911, *GFI* = 0.913, *RMSEA* = 0.065. These findings follow the same structure in both sample sets.

Male and female samples were compared for their relationship effects, which are summarized in Table 5. As a result of the findings, the level of information quality and brand love is significant and positive for both genders (male: β = 0.53, *t* = 5.45, *p* < 0.01; female: β = 0.46, *t* = 4.87, *p* < 0.01), accepting H6. The significant positive effect of system quality on brand love varies across gender (male: β = 0.49, *t* = 4.49, *p* < 0.01; female: β = 0.54, *t* = 5.69, *p* < 0.01), supporting H7. Similarly, virtual interactivity has a significant positive impact on brand-love across genders (male: β = 0.50, *t* = 4.52, *p* < 0.01; female: β = 0.46, *t* = 5.78, *p* < 0.01), accept H8. Finally, a positive relationship between rewards and brand love for both genders was found (male: β = 0.48, *t* = 3.69, *p* < 0.01; female: β = 0.47, *t* = 3.99, *p* < 0.01), not supporting H9. Therefore, instead of a reward, gender moderates the impact of community features on brand love.

**Table 5:** Hypotheses testing results across gender.

	**Hypotheses**	**Male**		**Female**		**Test results**
		**β**	***t*-values**	**β**	***t*-values**	
*H6*	Information quality → Brand love	0.53	5.45	0.46	4.87	Supported
*H7*	System quality → Brand love	0.49	4.49	0.54	5.69	Supported
*H8*	Virtual interactivity → Brand love	0.50	4.52	0.46	5.78	Supported
*H9*	Reward → Brand love	0.48	3.69	0.47	3.99	Not supported

## Discussion

Social networking sites have extraordinarily changed the communication style of consumers and the hospitality sector worldwide. Consumers have become sharp in making conversation with the hotel through an online platform overall and brand communities specifically. Developing and maintaining a brand lover client base in such a digitalized era is a big obstacle that hospitality faces. Hotels industries are more attentive to recognizing higher brand love drivers than properly used marketing notions like perceived service quality and customer satisfaction. Brand love has been proposed to be a potential unexpected predictor for eWOM. Thus, hospitality industries are emerging brand communities on the social network to communicate, stimulate, and advertise their offering to their consumers.

Our model provides insight into the importance of information quality, system quality, virtual attractively, and reward as critical drivers of brand love. Our findings suggest that managers should focus their marketing communications on improving brand attributes for consumers, which will positively affect consumer brand estimations. Our findings imply that managers must focus on expressing their brand-related (organizational) ethics to customers through SNS in a way that relates to these individuals' personal (ideal) brand love while retaining the brand's (organization's) fundamental values to create a distinct proposing.

This study established the possible effects of online community characteristics such as information quality, system quality, virtual interactivity, and rewards on brand love and the subsequent impacts of brand love on eWOM. All characteristics positively influence brand love, with information quality and system quality manner the decisive influence. Brand love also has a progressive impact on eWOM. This study adds brand love literature by offering an empirically confirming a unique framework for brand lovers with the hospitality community on SNS, applying the moderating role of gender.

Investigating the role of gender allows marketers to determine whether they want gender-based strategies implemented by men and women. In this study, moderation analysis reveals that the effect of online brand community characteristics on brand love is persistent across men and women. This means that the gender breach in online platforms is increasing. Previous research has shown that the system quality is most influencing factor effects on male than female. In contrast, the female content quality is observed as significant (Chu and Sung, 2015), and women report information quality is less than men (Liu et al., 2018). However, this study's finding is constant with current studies that established the gender difference in the online context, mostly regarding information quality, system quality, and virtual interactivity instead of reward (Mishra et al., 2018). Likewise, this study's result is consistent with Sohaib et al. (2019), who show that the gender hole in the online platform is increasing.

### Theoretical Implications

Our research adds concepts in the fields of information sorting and service management. This study extends the literature by (a) expanding our view of the role of information quality, system quality, virtual interactivity, and rewards effects as key drivers of brand love in the hospitality sector; (b) the effect of brand love on eWOM; (c) the role of eWOM in forming eventual consumer brand love in; and (d) exploring the role of gender as a potential moderator in the information quality, system quality, virtual interactivity, and reward. Theoretically, our model study emphasizes the stated effects of online information on brand love, which is evidently lacking in the literature. Whereas, the previous research has primarily examined brand love through the prism of relationship marketing or S-D logic (Vargo and Lusch, 2017), Little is revealed regarding the impact of eWOM motivator factors and brand love through the setting of attribution theory, as reported in this research article. As a result, our adoption of an integrative attribution theory perspective supplements current findings and supports the conceptual claims of these theories in the online information setting.

Second, even as studies on deception in a variety of many online settings have been conducted, the interpretation of reviewers with brand love and consumers' belief in the authenticity of reviewers' inference gestures has received less theoretical attention. Studies on the link between online reviews and brand love have been done in a limited number of cases (Ahmad and Sun, 2018; Hayat Bhatti et al., 2019). In contrast to previous research, our study adds to prior knowledge about eWOM sharing by the potential consumer on SNS. Hotel customers rely on experienced consumers to purchase and use a specific brand product or service, and our findings show that potential consumers posted on hotel and travel websites are influenced by hotel customers.

Third, whereas gender is a well-reported variable in the literature, little study has been conducted to evaluate the existence of gender effects on customer engagement. To assess whether or not, marketers might gain from gender-specific segmentation methods in the service sector of online review, we investigated the moderating influence of gender on the connection between information quality, system quality, virtual interactivity, and rewards. Interestingly, we discovered that gender significantly affected this connection instead of reward, adding an additional theoretical contribution to this work.

Fourth, by focusing on a specific sort of online hotel reviewer, the current study contributes to the body of knowledge in information processing. Our findings contradict Ahmad and Sun (2018) and Xie et al. (2011), who found that online information and sharing of consumers' experiences build brand trust. In comparison, the current study findings imply that consumers rely on confirmed and verified results of hotel industry experts' products and services because they believe online reviews provided by experienced consumers are accurate and dependable. We also discovered that consumers use online reviews from previous customers as a source of credibility when making hotel reservations.

### Managerial Implications

This study has a variety of managerial implications for hotel management marketing strategy. In the electronic era, one-to-one marketing and user-generated (including posting information) content are becoming crucially influential for the strategic improvement of consumer eWOM sharing and brand dedication (Brodie et al., 2011). In this setting, information quality, system quality, and virtual interaction are critical in promoting consumer motivations to enter and attach to a specific brand.

The functionality of online brand communities has improved recently, allowing marketers to encourage brand lovers that strengthen to share eWOM. According to the findings, the most influential predictors of brand lovers in online brand communities are information quality and virtual interactivity. As a result, organizations are advised to provide relevant, reliable, and efficient services, generate key messages, encourage the user to engage with the brand and other customers, allow consumers to request questions, submit inquiries, and exchange ideas and information. Marketers must set up quality control structures to confirm the quality of information. For example, at the start of reviews, hotel and hotel booking review websites may upload a photo of the peer reviewer's hotel booking voucher as well as a picture of him or her in the hotel room.

The study's findings indicate that to improve system quality and increase virtual interactivity, and the hotel industry could enhance the effectiveness of information search by providing superior navigational options, tracking members' previous browsing data, and proposing or emphasizing the best popular matters presently being imparted. This could result in noticeable effects that entice customers to stay with a specific brand. Marketers must give a more structured approach for customers to publish, share, and participate in brand community discussions in their entirety. This may also encourage more consumers to participate and contribute. To investigate the reviews on the hotel website and SNS, the hotel's manager could create an organization that registers industry specialists and makes their knowledge available on a separate page of their sites as well as on SNS. This will assist hotel organizations in developing a strong brand community through SNS.

### Limitation and Future Direction

In addition to making contributions to both theory and practice, our research has some limitations that can be applied to future studies. First, the developed framework was tested in the context of virtual brand communities, which was a unique setting. As a result, future researchers may be concerned about investigating the framework in offline settings or exploring its dynamics in composite online/offline platforms (Hollebeek et al., 2019). Second, our research examined eWOM as a direct result of brand love. However, additional dependent variables may comprise the consumer's desire to pay a premium for specific offerings, service innovation, and brand image. Third, the participants for this study were all from Beijing. Future researchers can resemble the study using different countries and markets to test for the effects of variances in market situations and cultures and simplify our findings and model. In addition, because the research used cross-sectional data, future researchers may be able to use longitudinal data to confirm the causal mechanisms identified herein. Finally, the scope of the study is restricted to the hospitality industry. Future studies can test our model's robustness against other sectors, such as healthcare, banking, and airline, to see if it holds up in different situations.

## Data Availability Statement

The raw data supporting the conclusions of this article will be made available by the authors, without undue reservation.

## Ethics Statement

The studies involving human participants were reviewed and approved by Anhui Polytechnic University. The patients/participants provided their written informed consent to participate in this study.

## Author Contributions

Conceptualization and data analysis: MB and UA. Methodology: YZ and MB. Writing—original draft: MB and SC. Writing—review and editing: UA and YZ. Formatting, data analysis, and literature review: ZW. All authors have read and agreed to the published version of the manuscript.

## Funding

This work was supported by the National Natural Science Foundation of China (grant number 72071002) and the Young Program of Natural Science Foundation of Anhui Province, China (grant number 1708085QG168).

## Conflict of Interest

The authors declare that the research was conducted in the absence of any commercial or financial relationships that could be construed as a potential conflict of interest.

## Publisher's Note

All claims expressed in this article are solely those of the authors and do not necessarily represent those of their affiliated organizations, or those of the publisher, the editors and the reviewers. Any product that may be evaluated in this article, or claim that may be made by its manufacturer, is not guaranteed or endorsed by the publisher.
